# Production of leafy biomass using temporary immersion bioreactors: an alternative platform to express proteins in transplastomic plants with drastic phenotypes

**DOI:** 10.1007/s00425-012-1829-1

**Published:** 2012-12-22

**Authors:** Franck Michoux, Niaz Ahmad, Anna Hennig, Peter J. Nixon, Heribert Warzecha

**Affiliations:** 1 Alkion Biopharma SAS, 4 rue Pierre Fontaine, 91058 Evry, France; 2 Division of Molecular Biosciences, Sir Ernst Chain Building-Wolfson Laboratories, Imperial College London, South Kensington Campus, London, SW7 2AZ UK; 3 Present Address: Agricultural Biotechnology Division, National Institute for Biotechnology and Genetic Engineering, Jhang Road, Faisalabad, Pakistan; 4 Faculty of Biology, Plant Biotechnology and Metabolic Engineering, Technische Universität Darmstadt, 64287 Darmstadt, Germany

**Keywords:** Chloroplast transformation, Containment, Leafy biomass, Temporary immersion bioreactor

## Abstract

Chloroplast transformation technology is a promising approach for the production of foreign proteins in plants with expression levels of up to 70 % of total soluble protein (TSP) achieved in tobacco. However, expression of foreign protein in the chloroplast can lead to drastic or even lethal effects in transplastomic plants grown in soil, thereby potentially limiting the applicability of this technology. For instance, previous attempts to express the outer surface protein A (OspA) from *Borrelia burgdorferi* in tobacco chloroplasts led to plant death when expressed at 10 % TSP. We show here that this earlier transplastomic line, as well as a new plant line, OspA:YFP, expressing OspA fused to the yellow fluorescent protein, can be propagated in temporary immersion bioreactors (TIBs) using AlkaBurst™ technology to produce leafy biomass that expressed OspA at levels of up to 7.6 % TSP, to give a maximum yield of OspA of about 108 mg/L. Our results show that TIBs provide an alternative method for the production of transplastomic biomass expressing proteins toxic for plants and is a particularly useful approach when ‘absolute’ containment is required.

## Introduction

As plastids are inherited maternally in most crop plants (Birky 1995), chloroplast transformation has been advocated as an environmentally friendly approach to produce transgenic plants (see Bock and Warzecha 2010 for review). Problems associated with nuclear transformation such as gene silencing, epigenetic effects and variable gene expression have not been observed after transforming the chloroplast genome. High-level expression of transgenes inserted into the chloroplast genome, or plastome, is also favoured by the high degree of polyploidy, with copy numbers of up to 20,000 per cell (Bock 2007). Numerous studies have shown that chloroplasts have a remarkable capacity to express foreign proteins, with levels of up to 70 % of total soluble protein (TSP) achieved (Oey et al. 2009; Ruhlman et al. 2010), which is generally a much higher value than that obtained by nuclear transformation, although some notable exceptions have been reported (Verwoerd et al. 1995; De Jaeger et al. 2002).

However, high expression levels can cause toxic effects to the host plant, leading to reduced growth and even premature death. For example, expression of tetanus toxin fragment C (TetC) in tobacco chloroplasts can be detrimental to transplastomic plants, which could only survive after grafting onto wild-type root stocks (Tregoning et al. 2003). Similarly, the massive expression of phage lytic protein, PlyGBS, imposed a metabolic burden on the host plants (Oey et al. 2009). Growth retardation was also observed when transplastomic expression of maltose-binding protein (MBP) reached 37 % of TSP, possibly due to impaired export of maltose from the chloroplast (Ahmad et al. 2012a). Expression of a thylakoid membrane protein, the plastid/plastoquinol terminal oxidase (PTOX) from *Chlamydomonas reinhardtii*, within tobacco chloroplasts rendered plants sensitive to high light (Ahmad et al. 2012b). Overall the current data suggest that the toxic effects of foreign proteins accumulating within photosynthetically active plastids might pose a strong limitation for this technology.

Previously, we have shown that healthy transplastomic plant biomass expressing high quantities of recombinant protein could be propagated efficiently using temporary immersion bioreactors (TIBs) (Michoux et al. 2011). To strengthen this technology further, we have tested whether high-level heterologous protein expression could be achieved using tobacco transplastomic lines that showed a lethal or impaired phenotype when grown in soil, in our case due to the expression of a bacterial outer surface protein A (OspA) from *Borrelia burgdorferi* in tobacco chloroplasts. OspA is a potent vaccine antigen used for the prevention of Lyme disease, a multisystem inflammatory condition prevailing in the Northern hemisphere (Hennig et al. 2007). When a full-length non-lipidated mutant version of OspA was produced within tobacco chloroplasts (in line OA14) at levels of 10 % TSP, its accumulation disrupted photosynthesis and the host plants could not grow autotrophically (Hennig et al. 2007). Here we have used an immunoblotting approach to examine the accumulation of OspA in cell-suspension cultures and in transplastomic plant biomass grown using TIBs, both in the original OA14 line, as well as a second tobacco line, OA:YFP, expressing a yellow fluorescent protein (YFP)-tagged derivative, which was created with the ultimate aim to visualise the location of OspA in the chloroplast. Collectively, our results show that growing plants in TIBs can be an effective technology to limit the unwanted ‘side effects’ of toxic proteins, thereby enabling the large-scale production of difficult targets.

## Materials and methods

### Plant material and growth of plant biomass in temporary immersion bioreactors

*Nicotiana tabacum* cv Petit Havana was used in this study. Calli of wild-type as well as transplastomic shoots (OspA seeds provided by Prof. Warzecha) were produced by placing small leaf discs on callus induction medium (CIM), which is Murashige and Skoog medium (MS) supplemented with 1 mg/L of 1-naphthaleneacetic acid (NAA) and 0.1 mg/L kinetin (K). Cell suspensions were generated by incubating 5 g of callus per litre of CIM lacking the agar at a constant agitation of 140 rpm. Wild-type, OA14 and OA:YFP biomass were generated in TIBs as described previously (Michoux et al. 2011) and using the AlkaBurst™ technology. In more detail, 1 g/L of cell suspension was used as inoculum and the immersion time was set for 4 min every 8 h. Seedlings, calli, cell suspensions and TIBs were grown at 25 °C, under a 16-h photoperiod (at 50 μmol photons m^−2^ s^−1^) at 30 % humidity in a Fi-Totron 600H incubator (Sanyo, Watford, UK). The biomass was harvested after a 40-day period, and was dried in an oven overnight at 80 °C after recording fresh weight to determine dry weight.

### Vector construction, transformation, and regeneration of transplastomic plants

For cloning of the *ospA*:*yfp* fusion for chloroplast expression, the full-length *ospA* sequence was amplified from pNT2-OspA (Glenz et al. 2006) using primers POspAforNcoI (5′-TTACCATGGAAAAATATTTATTGGGAATAGG-3′) and POA211 (5′-TGCTCGAGTTTTAAAGCGTTTTTAATTTCATCAAG-3′) adding *Nco*I and *Xho*I restriction sites to the 5′- and 3′-end, respectively. The sequence coding for YFP was amplified from pEYFP (Clontech, USA) using primers PYFP105 (5′-ATCTCGAGATGGTGAGCAAGGGCGAGG-3′) and PY1.1rev (5′-AGTTTCTAGAGAGCTCTTAGTGATGGTGATGGTGATGCTTG-3′) adding *Xho*I and *Xba*I restriction sites. PCR products were cloned into vector pCRblunt (Invitrogen, Karlsruhe, Germany) and resulting vectors pOA1432 and pYFP1433 were sequenced. Inserts were isolated as *Nco*I*/Xho*I fragment in case of pOA1432 and *Xho*I*/Xba*I fragment in case of pYFP1433 and used for a triple ligation with vector pKG27 (Glenz et al. 2006) which was cleaved *Nco*I*/Xba*I prior to ligation. The 1.3 kb expression cassette was inserted as *Sma*I*/Hin*dIII fragment into the chloroplast expression vector pRB95 (Ruf et al. 2001). The resulting vector was termed pOA:YFP4411.

Transformation of *Nicotiana tabacum* Petit Havana and regeneration were carried out as described previously (Hennig et al. 2007). The primary shoots were screened by PCR (data not shown) and the positive transformants were maintained for three rounds of selection to reach homoplastomy. Integration of the transgenes as well as homoplastomy of the transformants was assessed by restriction fragment length polymorphism (RFLP) as described previously (Hennig et al. 2007). Three independent plant lines were analysed. Tobacco line OA14 used in this study was initially described by Hennig et al. (2007).

### Protein extraction and quantification

Extraction of soluble proteins from transplastomic plant cells as well as from leafy biomass produced in TIBs, their analysis by SDS-PAGE and subsequent immunoblotting were carried out as described by Hennig et al. (2007).

## Results and discussion

### Generation of homoplastomic plants expressing OspA-YFP

In a previous study, a tobacco plant line, OA14, was generated expressing a mutant version of OspA unable to attach to lipid due to an altered amino acid: Gly (GGT) instead of Cys (TGT) at position 17 (Hennig et al. 2007). Plants producing either native lipidated OspA or non-lipidated OspA could only be propagated in vitro on sugar containing media but rapidly died when plants were transferred to soil. Most likely, accumulation of the recombinant OspA within the thylakoid membrane system together with its extraordinary stability against proteolytic degradation interferes with photosynthetic electron transport and abolishes photoautotrophic growth when a certain threshold level of protein is reached. In contrast, plant lines accumulating OspA at about 1 % TSP show minimal growth retardation and develop normally (Glenz et al. 2006).

**Fig. 1:**
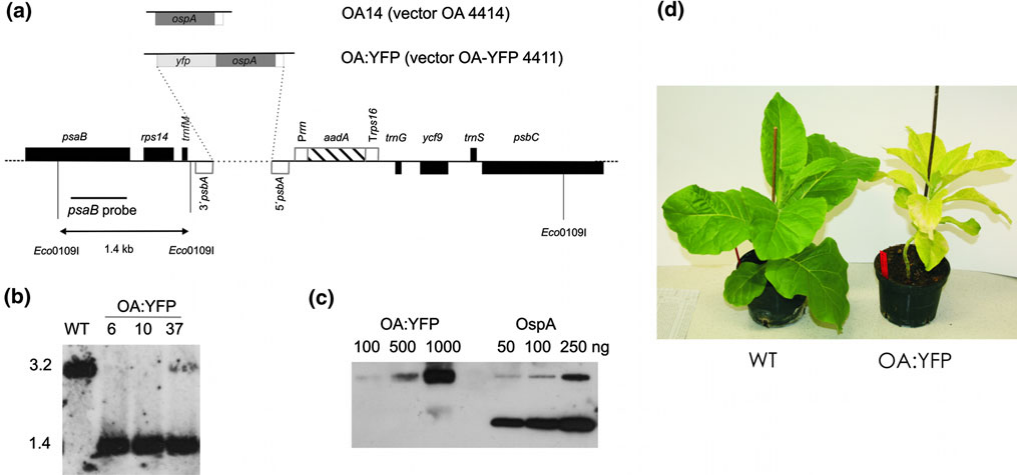
Generation of homoplastomic plants expressing *OspA:YFP* fusion protein. **a** Sequences coding for *YFP* were fused with the one coding for *OspA* and placed between *psbA* 5′- and 3′-untranslated regions and subsequently cloned into the pRB95 vector (Ruf et al. 2001) to integrate the transgenes between *trnfM* and *trnG* of the tobacco plastomic region. **b** Southern blot analysis of plants to confirm the correct integration of transgenes at the chosen site and prove homoplastomy. Genomic DNA was isolated, digested with *Eco01091* and hybridized with a digoxigenin-labelled probe corresponding to the flanking region of the plastome amplified from wild type. Fragment sizes shown are in kb. **c** Immunoblot analysis of proteins from *OA14:YFP* transplastomic plants carried out using an anti-OspA antibody. Recombinant *OspA* purified from *E. coli* was used as standards for quantification. Amounts of total protein loaded indicated. The level of OspA-YFP fusion expressed in 1,000 ng of TSP was equivalent to approximately 50 ng of the OspA standard. **d** Phenotype of plants expressing OspA-YFP fusion protein compared to wild type

In order to help localize OspA in future experiments, a YFP-tagged version, OA:YFP, was constructed (Fig. 1a) and expressed in tobacco chloroplasts. Southern blot analysis confirmed the integration of the OA:YFP encoding sequences at the required site in the plastome as well as the homoplastomic nature of the transformants (Fig. 1b). In contrast to OA14 (Hennig et al. 2007), OA:YFP was now able to grow in soil in the greenhouse, but was noticeably more yellow than the WT (Fig. 1d) and hardly set seed. Similar pale and retarded growth phenotypes have also been observed when the plastoquinol/plastid terminal oxidase from *Chlamydomonas reinhardtii* was expressed in tobacco chloroplasts and have been linked to enhanced susceptibility to chronic photoinhibition (Ahmad et al. 2012b).

In order to quantify the accumulation of the OspA-YFP fusion, TSP was extracted and analysed by SDS-PAGE followed by immunoblotting using an anti-OspA antibody. The level of expression was quantified by comparing signal intensities with purified recombinant OspA standards produced in *E. coli*. The results suggested that the OA-YFP was expressed in leaves at a level of approximately 5 % TSP (Fig. 1c), whereas OspA alone was shown in previous work to be expressed at higher levels (10 % TSP, Hennig et al. 2007). The presence of a faint upper band in the OspA standards in Fig. 1c is due to the aggregation of lipophilic OspA (Hennig et al. 2007). The less pronounced accumulation of OA-YFP compared to OA14 could be one of the reasons for the less-severe phenotype. A concentration-dependent effect of protein accumulation on plant growth was also observed when glutathione-*S*-transferase (GST) was expressed in tobacco chloroplasts. High levels of GST expression caused aberrant pollen development thereby inducing cytoplasmic male sterility in the host plants (Ahmad et al. 2012a), whereas lower levels of GST expression had no such effect (Le Martret et al. 2011).

### Production of transplastomic biomass in temporary immersion bioreactors and OspA accumulation

**Fig. 2:**
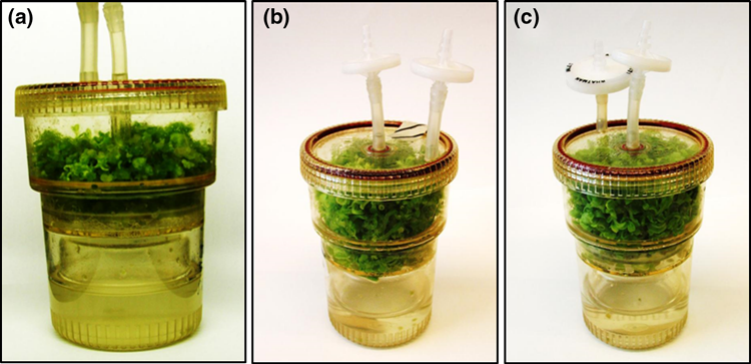
Growth of transplastomic leafy biomass expressing OspA in TIBs. Transplastomic biomass of OA14 (**a**), OA:YFP (**b**) and wild type (**c**) after a 40-day inoculation period in TIBs supplemented with AlkaBurst™

The availability of the two lines expressing OspA, one toxic for plant growth (OA14) and one impaired but viable (OA:YFP), allowed for a direct comparison of their accumulation of OspA in various potential expression systems. Both the wild-type as well as transplastomic shoots were used to raise calli to generate the corresponding cell-suspension cultures. These cells were then transferred to TIBs to produce transplastomic biomass using AlkaBurst™ technology. After 40 days, the cells had successfully developed into healthy green leafy shoots in all lines analysed (OA14 line, Fig. 2a; OA:YFP line, Fig. 2b and wild type, Fig. 2c). The fresh and dry weight of the OA:YFP line was comparable to that of wild type, with growth of the OA14 line only slightly less (Table 1).

**Table 1:** Comparison of the transplastomic biomass produced through the temporary immersion bioreactor system

Plant line	Fresh weight (g/L)	Dry weight (g/L)
Nt–Wt	261.8 ± 21.9	10.7 ± 0.7
OA:YFP	253.4 ± 35.8	10.6 ± 0.8
OA14	191.1 ± 26.8	8.3 ± 0.5

Values represent the average of three bioreactors for each line (*n* = 3), along with respective standard deviations

Semi-quantitative immunoblotting indicated that the expression levels of OspA (7.6 % TSP) and OspA:YFP (1.1 % TSP) were lower in TIB-grown biomass compared to the plant leaves, at 10 and 5 % of TSP, respectively (Fig. 1c). One possible factor for the lower accumulation of OspA and OspA:YFP in TIB-grown biomass could be related to poorer light penetration into the centre of the bioreactors, which might have affected the development of fully functional chloroplasts. Light penetration is also important for expression of the *oa14* and *oa:yfp* transgenes, which are both under the control of the light-driven *psbA* promoter (Klein and Mullet 1987). Michoux et al. (2011) have been able to increase expression of GFP+ from 0.5 to 4 % TSP by simply changing light intensity and sucrose content. Thus, further optimisation might lead to improved production of OspA using the TIB system.

When comparing the cell suspension and TIBs, and the two different versions of OspA, the level of expression is much higher in the case of TIBs. For example, the expression of OspA-YFP in TIB-grown biomass was 27 times higher when measured as TSP, 79 times higher in case of fresh weight (FW), 72 times higher in case of dry weight (DW) and 45 times higher per volume basis than the cell suspensions (Table 2). Similarly, the expression of OspA was observed to be about 12 times higher when measured as TSP, 51 times higher in case of FW, 48 in case of DW and 22 higher when the biomass was produced in TIBs rather than as cell suspensions, when comparing on an equal volume basis (Table 2). Qualitatively similar results were obtained when GFP+ and a tetanus toxin fragment C (TetC) expressing biomass was produced in cell suspension and TIBs (Michoux et al. 2011). The levels of GFP+ and TetC were in the range of 0.5–0.8 % of TSP, respectively, when biomass was produced in the cell suspension, whereas, the expression levels reached 33 % TSP in case of GFP+ and 8 % TSP in case of TetC when the biomass was produced through the TIB system (Michoux et al. 2011).

**Table 2:** Quantification of OspA levels in transplastomic biomass produced by cell suspension (cells) and temporary immersion bioreactor system (TIBs)

Plant Line	% TSP	mg/g FW	mg/g DW	mg/L
OA:YFP—Cells	0.04	0.001 ± 0.0002	0.024 ± 0.005	0.44 ± 0.09
OA:YFP—TIB	1.10	0.079 ± 0.011	1.726 ± 0.068	20.02 ± 2.83
OA14—Cells	0.60	0.011 ± 0.002	0.268 ± 0.011	4.89 ± 0.55
OA14—TIB	7.60	0.568 ± 0.012	13.065 ± 0.276	108.54 ± 15.22

Values represent the average of three bioreactors for each line (*n* = 3), along with respective standard deviations

The possible reason for higher accumulation of recombinant protein in TIB-grown biomass compared to cell-suspension cultures could be due to differences in the number of plastids per cell, the impaired development of the chloroplast in the cell-suspension culture and differences in the activity of the promoter used to drive expression of the transgenes. Silhavy and Maliga (1998) observed that the activity of 16S rRNA promoter in rice embryogenic cells was around sevenfold less than that observed in the leaves. Likewise, it remains possible that the activity of the light-regulated *psbA* promoter used in this study was down-regulated in the cell-suspension culture, leading to reduced protein expression. It will be of great interest to test the expression levels of such transplastomic lines in photoautotrophic suspension cultures, where expression levels could be higher than in classical plant cell cultures (Hampp et al. 2012).

**Fig. 3:**
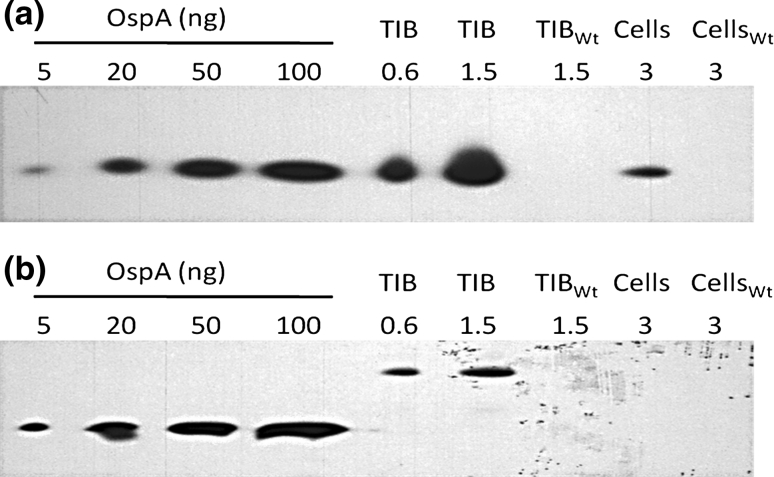
Accumulation of *OspA* in transplastomic rootless biomass and its quantification. Total soluble protein was extracted from OA14 (**a**) and OA:YFP (**b**) grown as a cell-suspension culture (*Cells*) or in a *TIB* and subjected to SDS-PAGE followed by immunoblotting. Samples from WT (*Cells*_*Wt*_ and *TIB*_*Wt*_) were used as controls. Different amounts of purified *OspA* from *E. coli* were used as standards

In all cases, the expression of YFP-tagged OspA was much lower than the OspA alone (Table 2; Fig. 3). Various studies have shown that the expression of foreign proteins is dependent on the choice of promoter and the 5′-untranslated region of the mRNA (see Maliga 2003 for review). However, as the same expression elements were used to drive expression of both OspA and OspA-YFP, the difference in expression must be related to other factors, yet to be determined.

In summary, we have shown that the propagation of transplastomic biomass using TIBs is potentially a useful technology to propagate plant material expressing proteins toxic to the host plant. The use of TIBs also ensures absolute containment of transgenic material and so could be successfully applied to large-scale production of high-value targets in plants without going into the field.

## Abbreviations

TSP: Total soluble protein
OspA: Outer surface protein A
YFP: Yellow fluorescent protein
TIBs: Temporary immersion bioreactors
RFLP: Restriction fragment length polymorphism
